# Changes in sensor recorded activity patterns and neuropsychiatric symptoms after deep brain stimulation for Parkinson’s disease: 5 case reports

**DOI:** 10.1186/s12883-025-04030-w

**Published:** 2025-01-17

**Authors:** Lena C. Bruhin, Michael Single, Aileen C. Naef, Katrin Petermann, Mario Sousa, Matilde Castelli, Ines Debove, Marie E. Maradan-Gachet, Andreia D. Magalhães, Andreas A. Diamantaras, M. Lenard Lachenmayer, Gerd Tinkhauser, Julia Waskönig, Christopher M. el Achkar, Alia Lemkaddem, Mathieu Lemay, Paul Krack, Tobias Nef, Deborah Amstutz

**Affiliations:** 1 ARTORG Center for Biomedical Engineering Research, University of Bern, Bern, Switzerland; 2 Department of Neurology, University Hospital Bern, Inselspital, University of Bern, Bern, Switzerland; 3 Graduate School for Health Sciences, University of Bern, Bern, Switzerland; 4 Centre Suisse d’Electronique et de Microtechnique, Neuchâtel, Switzerland

**Keywords:** Parkinson’s disease, Deep brain stimulation, Neuropsychiatric symptoms, Sensor technology, Objective assessment

## Abstract

**Background:**

Effects of subthalamic nucleus deep brain stimulation (STN-DBS) on neuropsychiatric symptoms of Parkinson’s disease (PD) remain debated. Sensor technology might help to objectively assess behavioural changes after STN-DBS.

**Case presentation:**

5 PD patients were assessed 1 before and 5 months after STN-DBS with the Movement Disorders Society Unified Parkinson’s Disease Rating Scale part III in the medication ON (plus postoperatively stimulation ON) condition, the Montreal Cognitive Assessment, the Questionnaire for Impulsive-Compulsive Behaviors in Parkinson’s Disease Rating Scale present version, the Hospital Anxiety and Depression Scale and the Starkstein Apathy Scale. Steps taken per hour, nighttime spent in bed and time spent outside were monitored with a smartwatch and ambient sensors placed in patient homes for an average of 20 days pre- and postoperatively. Postoperative improvement in ICDs and concomitant anxious-depressive symptoms was observed in 3 patients and was accompanied by a decrease in steps taken per hour, as well as an increase in nighttime spent in bed. In the two patients without baseline ICDs, mild anxiety and apathy improved postoperatively, and no new neuropsychiatric symptoms occurred. Steps taken per hour did not decrease in these cases and nighttime spent in bed improved in one of the patients, but decreased in the other, who had experienced pain during OFF-phases at night before STN-DBS.

**Conclusion:**

Changes in neuropsychiatric symptoms are associated with distinct activity patterns after STN-DBS, and wearable and ambient sensors may aid to capture those gradual shifts in behavior.

**Supplementary Information:**

The online version contains supplementary material available at 10.1186/s12883-025-04030-w.

## Background

Parkinson’s disease (PD) is the second most common neurodegenerative disease and is defined by hallmark motor symptoms including bradykinesia, rigidity, tremor and postural instability [1, 2]. Neuropsychiatric symptoms such as depressive symptoms, anxiety, apathy, impulse control disorders (ICDs), and cognitive decline have a high prevalence in PD and negatively impact quality of life of affected individuals [3, 4]. Nevertheless, these neuropsychiatric symptoms are under recognized and undertreated [4, 5]. While depression, anxiety and apathy have been linked to a lack of dopamine, ICDs have been associated with an overstimulation of mesolimbic pathways caused by dopaminergic medications used to treat PD [6–8]. Patients with advanced PD often suffer from motor and neuropsychiatric fluctuations. At the peak dose of the dopaminergic medication, they experience dyskinesia, euphoria and impulse control disorders, whilst during wearing off of dopaminergic medication, bradykinesia, rigidity, tremor, anxiety, depressive symptoms and apathy prevail [9]. Subthalamic nucleus deep brain stimulation (STN-DBS) is a safe and effective treatment for the motor fluctuations and dyskinesia associated with advanced PD [10]. However, its effects on neuropsychiatric symptoms remain debated. Some studies report an improvement of ICDs and depressive symptoms [11, 12], whilst others describe stimulation-induced hypomania [13], motor impulsivity [14–16] and postoperative apathy [17]. To assess neuropsychiatric symptoms, clinicians must rely on reports of patients and caregivers, which can be inaccurate due to a lack of insight for gradual changes in behavior [18]. A potential way of overcoming this issue in the future is the additional use of sensor technology to assess behavioral changes. Studies have proven that wearable and ambient sensors can objectively assess motor symptoms in PD and that long-term monitoring of activity patterns in subjects with neurodegenerative disease is possible [19, 20]. The aim of this case series was to evaluate whether wearable and ambient sensors can aid in capturing changes in neuropsychiatric symptoms before and after STN-DBS.

## Case presentations

### Case selection

Five patients with a diagnosis of idiopathic PD [2] and planned STN-DBS surgery were recruited at the Movement Disorders Centre at the University Hospital of Bern within the framework of a larger study (KEK: 2020 − 01777). Written consent was obtained from all participants in accordance with the Declaration of Helsinki. The cases were selected so that different motor (tremor-dominant, akinetic-rigid and mixed PD) and neuropsychiatric (patients with ICDs versus patients without ICDs) profiles could be studied. Selection criteria for STN-DBS and surgical procedures have previously been described in detail [21]. Participants underwent several experimental procedures, and sensor recordings were performed for an average duration of 19.2±4.3 days at 31.6±13.9 days before and 20.8±7.1 days at 161.4±19.7 days after STN-DBS. The procedures relevant to this case series are listed below.

### Neurological and neuropsychiatric assessments

Motor symptom burden was assessed with the Movement Disorders Society Unified Parkinson’s Disease Rating Scale (MDS-UPDRS) [22] part III in the medication ON (plus postoperatively stimulation ON) condition. Levodopa equivalent daily dose (LEDD) and dopamine agonist (DA) daily dose were calculated. The neuropsychiatric assessment included the Montreal Cognitive Assessment (MoCA) [23], the Questionnaire for Impulsive-Compulsive Behaviors in Parkinson’s Disease Rating Scale (QUIP-RS) [24] present version, Hospital Anxiety and Depression Scale (HADS) [25] and the Starkstein Apathy Scale (SAS) [26].

### Sensor recordings

Sensor recordings consisted of a smartwatch and ambient sensors, which have previously shown good long-term acceptance by patients [27]. The smartwatch was worn on the wrist during waking hours. Contactless low-energy Bluetooth (BLE) ambient sensors were placed in participants homes and a pressure-sensitive bed sensor was placed under the mattress. All sensor recordings were performed simultaneously. For detailed information on models and recordings, see supplementary material.

Analyses were performed with Python 3.10 (Python Software Foundation, Wilmington USA). To visualize the neuropsychiatric profile of patients, spider plots were created for each patient. The maximum values of the spider plot axes were set to reflect the maximum scores obtained by the patients. Periods were classified as time spent outside the home when no ambient sensor BLE signal was detected by the smartwatch for more than 180 s. Based on this, the percentage of watch wear time spent outside was calculated. Nighttime spent in bed (7pm to 9am) was extracted from the user interface of the bed sensor. Daytime spent in bed was not analyzed, since periods spent in bed shorter than 2 h were recorded, but not saved in the user interface. Steps taken per hour were normalized with respect to the total watch wearing time.

### Group level outcomes

**Fig. 1:**
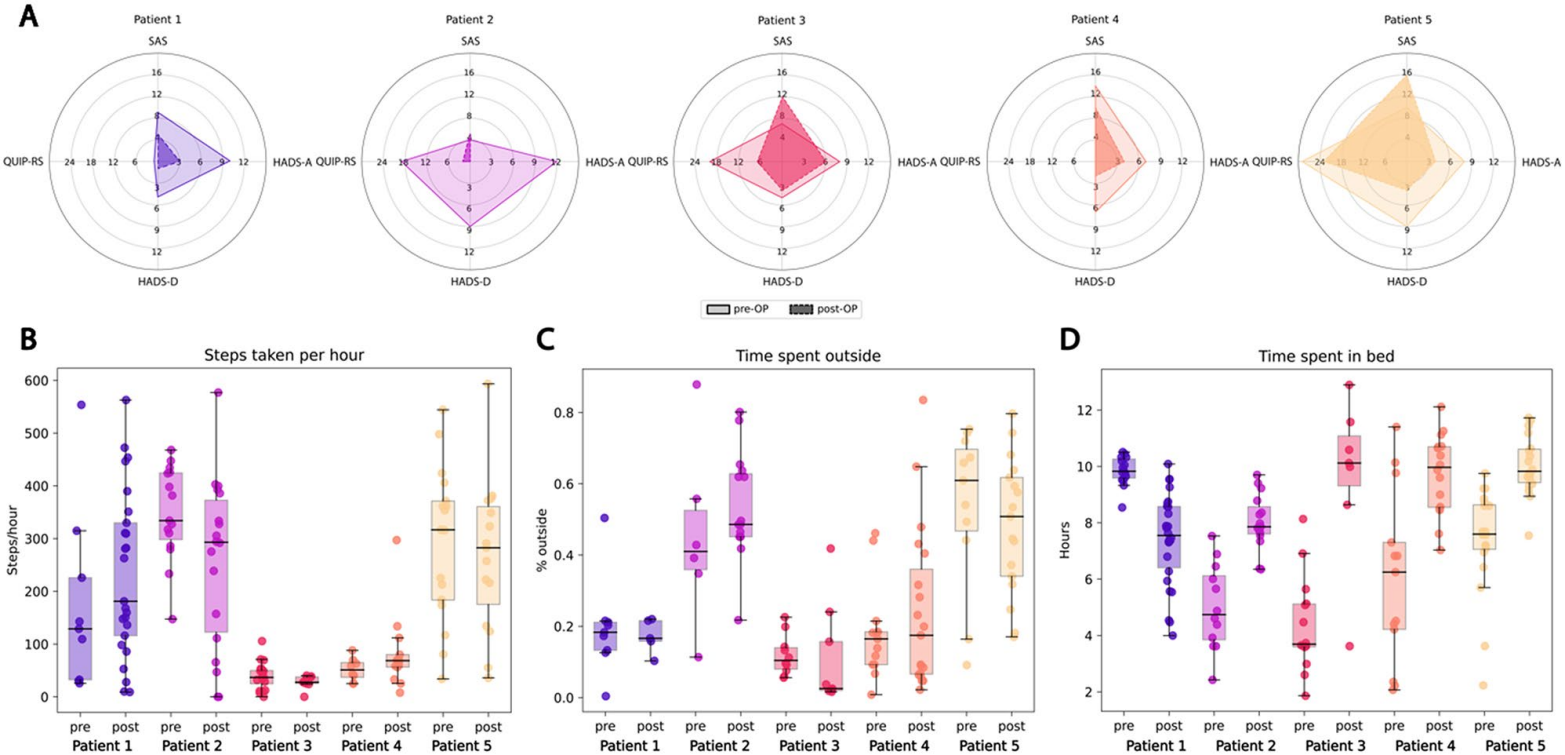
**(A)** Neuropsychiatric profiles of individual patients with the lighter area representing the baseline before DBS (deep brain stimulation) and the darker area follow-up with DBS; QUIP-RS = Questionnaire for Impulsive-Compulsive Disorders in Parkinson’s Disease Rating Scale present version; HADS-A = Hospital Anxiety and Depression Scale Anxiety Subscale; HADS-D = Hospital Anxiety and Depression Scale Depression Subscale; SAS = Starkstein Apathy Scale. (**B, C, D**) Activity profiles for each participant for steps taken per hour, time spent outside, and nighttime spent in bed

Baseline characteristics and group level results of the neurological and neuropsychiatric assessments are summarized in Table 1. Overall, motor symptom burden decreased after STN-DBS and dopaminergic medication was reduced. Anxiety, depressive symptoms, and ICDs improved. Figure 1 shows neuropsychiatric profiles and different activity measures for each patient.

**Table 1:** Demographic data and pre- and postoperative neurological, neuropsychiatric, and cognitive measures

	Baseline	Follow-up
n	5	5
Age	59.0 (± 9.9)	*
Gender (male/female)	1/4	*
Disease duration (years)	8.4 (± 1.8)	*
MDS-UPDRS part III	25.8 (± 13.9) (med-ON)	14.0 (± 6.7) (med-ON, stim-ON)
LEDD (mg/day)	1432.0 (± 624.6)	545.0 (± 253.5)
DA daily dose (mg/day)	212.0 (± 184.2)	80.0 (± 63.2)
SAS	8.8 (± 3.7)	9.6 (± 4.7)
HADS-A	9.0 (± 2.0)	3.4 (± 2.2)
HADS-D	7.0 (± 2.0)	2.2 (± 1.9)
QUIP-RS present	13.8 (± 12.8)	6.4 (± 9.7)
MoCA	27.6 (± 2.3)	27.2 (± 1.3)

Reported values except for gender are means (± standard deviation); LEDD = levodopa equivalent daily dose; DA = dopamine agonist daily dose; MDS-UPDRS III = Movement Disorders Society Unified Parkinson’s Disease Rating Scale part III assessed on medication at baseline and on medication plus stimulation at follow-up; HADS-A = Hospital Anxiety and Depression Scale Subscale Anxiety; HADS-D = Hospital Anxiety and Depression Scale Subscale Depression; QUIP-RS = Questionnaire for Impulsive-Compulsive Disorders in Parkinson’s Disease Rating Scale present version; MoCA = Montreal Cognitive Assessment

### Case 1

Patient 1 was a 70-year-old man with tremor-dominant PD and a disease duration of 7 years. Preoperatively, he had mild anxiety, which was characterized by worrying about daily activities, which had become difficult to perform due to his tremor. The rest of the neuropsychiatric scores and cognitive screening did not show any abnormalities (MoCA 29/30). He remained very active despite his tremor, enjoying home cooking and baking. He slept well at night. At follow-up, his anxiety improved (HADS-anxiety 10 to 3), and no new neuropsychiatric symptoms occurred. Motor symptoms improved and dopaminergic medication was reduced (LEDD 820 mg/d to 420 mg/d, MDS-UPDRS III 17 to 16). Nighttime spent in bed decreased (9.9±0.5 h to 7.4±1.6 h), steps taken per hour increased (206±166 to 236±151), and time spent outside remained relatively stable (19±13% to 17±4%).

### Case 2

Patient 2 was a 53-year-old man with akinetic-rigid PD and a disease duration of 10 years. Preoperatively, he had pathological ICDs, which were characterized mainly by excessive hobbyism and shopping. He spent his free time engaged with various projects (woodworking, fixing motorcycles, renovating his house), working day and night, sometimes using dangerous tools such as saws during OFF-phases. His mood was elevated, and he claimed he did not need more than a few hours of sleep. He reported anxiety and depressive symptoms associated with OFF-phases, but no apathy or impairment in cognitive screening (MoCA 30/30). At follow-up, neuropsychiatric symptoms improved (QUIP-RS 19 to 2, HADS-anxiety 12 to 0, HADS-depression 9 to 0) and were no longer pathological. Motor symptoms improved and dopaminergic medication was reduced (LEDD 1430 mg/d to 160 mg/d, MDS-UPDRS III 17 to 8). Nighttime spent in bed increased (5.00±1.5 h to 8.0±1.0 h), steps taken per hour decreased (346±84 steps to 257±140 steps) and time spent outside slightly increased (45±23% to 52±18%).

### Case 3

Patient 3 was a 63-year-old man with mixed akinetic-rigid and tremulous PD and a disease duration of 9 years. Preoperatively, he had pathological ICDs, which included binge eating and hobbyism (excessive computer use). He was feeling wide awake at night, spending his time on the computer and sleeping rarely more than a few hours. Furthermore, he reported mild anxiety symptoms and cognitive testing revealed mild cognitive impairment (MoCA 24/30). Postoperatively, ICDs and anxiety symptoms were no longer pathological (QUIP-RS 20 to 7, HADS-anxiety 8 to 6). Apathy increased (SAS 7 to 12) but remained under the cut-off. Motor symptoms improved and dopaminergic medication was reduced (LEDD 2400 mg/d to 650 mg/d, MDS-UPDRS III 50 to 21). Nighttime spent in bed increased (4.4±1.6 h to 9.6±2.8 h), steps taken per hour showed a tendency to decrease (39±24 to 28±14) and time spent outside remained stable (12±5% to 11±6%).

### Case 4

Patient 4 was a 64-year-old woman with akinetic-rigid PD and a disease duration of 10 years. Preoperatively, she had mild apathy, just below cut-off anxiety and depressive symptoms, but no ICDs or impairment in cognitive screening (MoCA 27/30). She reported a lack of energy and pleasure for everyday activities, feeling limited by her PD symptoms. At night, she experienced pain during OFF-phases. Postoperatively, apathy, anxiety and depressive symptoms decreased (SAS 14 to 10, HADS-anxiety 7 to 4, HADS-depression 7 to 2) and were below cut-off. Motor symptoms improved and dopaminergic medication was reduced (LEDD 950 mg/d to 740 mg/d, MDS-UPDRS III 25 to 19). Time spent in bed increased (6.0±3.0 h to 9.7±1.4 h), and steps taken per hour and time spent outside slightly increased (56±27 to 64±26; 18±13% to 24±22%).

### Case 5

Patient 5 was a 45-year-old man with akinetic-rigid PD and a disease duration of 6 years. Preoperatively, he had multiple ICDs, including pathological gambling in online casinos, excessive shopping, hobbyism with a newfound interest in painting, increased libido and appetite. He reported sleeping well, but getting up very early even on days that he did not go to work. He had mild anxiety and depressive symptoms associated with OFF-phases, but no apathy or impairment in cognitive screening (MoCA 28/30). At follow-up, anxiety and depressive symptoms improved and were below cut-off (HADS-anxiety 8 to 4, HADS-depression 9 to 4), ICDs improved but were still above cut-off (QUIP-RS 29 to 23) and mild apathy manifested (SAS 10 to 16). Motor symptoms improved and dopaminergic medication was reduced (LEDD 1560 mg/d to 755 mg/d, MDS-UPDRS III 20 to 6). Nighttime spent in bed increased (7.4±1.8 h to 10.0±1.0 h), steps taken per hour showed a tendency to decrease (282±130 to 268±140), and time spent outside slightly decreased (54±22% to 48±18%).

## Discussion

This case series evaluated the potential of sensor technology to record behavioral activity patterns associated with neuropsychiatric symptoms before and after STN-DBS.

The 5 presented cases revealed heterogeneous neuropsychiatric and activity profiles both before and after STN-DBS, stressing the importance of intra-individual comparisons. Interestingly, despite the motor improvement, not all patients showed an increase of steps taken per hour and time spent outside. In three patients, an improvement of baseline ICDs and anxious-depressive symptoms during medication OFF phases was associated with a decrease in steps taken per hour and a marked increase in time spent in bed during the night. Due to the design of the study, it is not possible to fully disentangle the distinct effect of anxiety, depression and ICDs on sensor measures. However, research shows that ICDs are associated with and increased drive during the day and nocturnal hyperactivity (decreased need for sleep with individuals pursuing ICD and related behaviors during the night) [28, 29]. For depression, the link relation with sleep is less clear, with some patients experiencing excessive sleeping as a symptom of depression and others suffering from insomnia [30]. As for overall activity, anxiety, depressive symptoms and apathy are associated with a decrease in goal-oriented behavior [29, 31]. For example, before STN-DBS, patient 2 showed an increased drive with hobbyism (sports, fixing motorcycles, and woodworking) and nocturnal hyperactivity even during off phases. Despite marked motor improvement after the operation, he spent significantly less time performing his hobbies and slept during the night rather than being active. Therefore, at least in the present three cases with preoperative ICDs, there seems to be a link between a postoperative improvement in ICDs, increase in time spent in bed (reflecting a normalization of nocturnal sleep) and decrease in steps taken per hour (reflecting a decrease in activity during the day). In the two patients without ICDs at baseline, mild anxiety and apathy improved postoperatively, and no new neuropsychiatric symptoms occurred. Contrary to the patients with preoperative ICDs, steps taken per hour did not decrease. Nighttime spent in bed improved in one of those patients, but decreased in the other, who had experienced pain at night associated with off phases which lead to insomnia before STN-DBS. Pain has been associated with sleep disturbances in PD [32], which highlights the importance of assessing pain.

At first glance, the finding that ICDs improve after STN-DBS might seem to contradict studies demonstrating that motor impulsivity is worse after STN-DBS [14–16]. However, other studies show that the impact of STN-DBS on motor inhibition depends on the stimulation site within the STN, with ventral STN leading to motor impulsivity and dorsal stimulation improving motor inhibition [33–37]. Importantly, motor impulsivity is only one facet of impulsivity, which is a multi-faceted construct, involving different cognitive processes, with STN-DBS showing different effects on different measures of impulsivity [38]. ICDs such as hypersexuality, binge eating, pathological gambling and shopping are not associated with motor impulsivity in inhibition tasks [39]. In the majority of available studies, ICDs show improvement after STN-DBS [40]. This decrease in ICDs is associated with a decrease in dopaminergic medications, which are known to be the biggest risk factor for ICDs [11, 40–42]. At the same time, a marked reduction of dopaminergic medication has been associated with apathy [11, 43, 44]. In the present five patients, dopaminergic medications were carefully tapered after DBS surgery to avoid postoperative apathy, with patients remaining on a low dose of levodopa and dopamine agonists. The observed improvement in depressive symptoms and anxiety is in line with meta-analyses showing an improvement in anxiety and depression following STN-DBS [45, 46]. Nevertheless, the follow-up with sensor recordings should be extended in further studies, since research shows that apathy may occur gradually over time due to dopaminergic desensitization [44, 47, 48].

In summary, case reports shows that especially the assessment of nocturnal behavior with a bed sensor is a promising measure for behavioral changes after STN-DBS, showing meaningful associations with neuropsychiatric symptoms, motor burden and dopaminergic medication. However, regarding the bed sensor, a limitation of this study is that due to the user interface, daytime naps or shorter sleep intervals during the night could not be analyzed. Furthermore, no detailed objective information on sleep parameters (e.g. time spent sleeping, different sleep stages) and reasons for poor sleep (e.g. hyperactivity versus pain) could be obtained. So far, monitoring specific aspects of asleep with sensor technology at home lacks reliability compared to polysomnography used in clinic [49, 50], but in the future technological advancements might help bridging this gap. Therefore, additionally to sleep sensors, interviews or scales to assess sleep quality and quantity should be used. Regarding the other sensors, steps taken per hour have also proved to be a useful measure in this study, whereas the time spent outside seemed to be less sensitive to change. Importantly, sensor technology allows to monitor gradual changes in behavior, which are often difficult to assess retrospectively in clinical practice. However, the interpretations of activity patterns should always take the context of the individual into consideration. To further explore the complex interplay between motor symptoms, neuropsychiatric symptoms, dopaminergic medications, STN-DBS, and their impact on activity and sleep patterns, studies with bigger patient numbers and more detailed statistical analyses, are needed.

In conclusion, this case series illustrates as a proof of principle that objective data provided by wearable and ambient sensors can be a useful addition to subjective answers provided by patients in questionnaires or interviews to evaluate and quantify gradual changes in behavioral activity patterns in PD, showing different STN-DBS outcomes in patients with different neuropsychological profiles. Objective assessment of behavioral activity with wearable sensors therefore shows potential to improve neuropsychiatric evaluation in the future.

## Electronic supplementary material

Below is the link to the electronic supplementary material.

Supplementary Material 1

## Data Availability

The datasets generated and/or analysed during the current study are not publicly available due to patient privacy concerns but are available from the corresponding author on reasonable request.

## Abbreviations

BLE: Low-energy Bluetooth
DA: Dopamine agonist
HADS: Hospital Anxiety and Depression Scale
ICD: Impulse Control Disorder
LEDD: Levodopa equivalent daily dose
MDS-UPDRS: Movement Disorders Society Unified Parkinson’s Disease Rating Scale
MoCA: Montreal Cognitive Assessment
PD: Parkinson’s Disease
QUIP-RS: Questionnaire for Impulsive-Compulsive Behaviors in Parkinson’s Disease Rating Scale
SAS: Starkstein Apathy Scale
STN-DBS: Subthalamic Nucleus Deep Brain Stimulation
