# Supplementary material for: Changes in sensor recorded activity patterns and neuropsychiatric symptoms after deep brain stimulation for Parkinson’s disease: 5 case reports
Source: BMC Neurol. 2025 Jan 17;25:25. doi: 10.1186/s12883-025-04030-w (PMC11740435; doi:10.1186/s12883-025-04030-w)
Supplement: Supplementary file 1 — Supplementary Material 1 [file 12883_2025_4030_MOESM1_ESM.docx]

# Supplementary material

**Sensor recording**

Sensor models are summarized in Table 1 and depicted in Figure 1 of the supplementary material below. An engineer placed all sensors in the patient homes and explained how to use the smartwatch. Patients were instructed to wear a smartwatch during waking hours, excluding shower- or bathtimes, and to charge it every night. The used smartwatch containes an accelerometer that measured movement as well as a Bluetooth module to communicate with the ambient sensors. Two different types of ambient sensors were used (bed sensor and BLE ambient sensors). The bed sensor was placed below the mattress at chest height and recorded movement and physiological parameters during sleep. This sensor is based on a piezoelectrical sensor and is CE certified. Bed presence periods of longer than 2 hours are saved on the server of EMFIT. If the patients left the bed for more than 15 minutes at a time, a new recording period started. Sleep data were downloaded onto the study servers every day. As for the BLE ambient sensors, five to seven of them were placed in the participants home, with the number of placed sensors depending on the layout of the living space. The following areas of the home were covered with sensors: Living-room, kitchen, bedroom, bathroom (if more than one major bathroom is in use, all of them are covered) and entrance area. In case of insufficient coverage in any other rooms, additional sensors were placed. The ambient sensors emit a BLE signal that is detected by the Bluetooth module of the smartwatch and detects the distance of the smartwatch wearing person to the BLE sensors. Therefore, only presence or absence of the participants was observed and not the presence of other family members. A base station consisting of a 4G-router, a small computer and a charging-station for all wearable sensors, were also placed in the patients’ home. This base station connected all ambient sensors and managed the secure data-upload to a data server, which is hosted in Switzerland. All collected data was uploaded automatically to the servers during the measurements and was monitored by an engineer. At the end of the measurement period a second home visit is conducted, where all the sensors are uninstalled again.

Table 1 supplementary material.

| Sensor | Model | Company | Sampling Frequency [Hz] |
| --- | --- | --- | --- |
| Smartwatch | PolarM600 | Polar Electro, Kempele FIN | 100 |
| Bed sensor | EMFIT QS | Emfit Ltd, Vaajakoski FIN | 0.25 |
| BLE beacon | iHomeLab Multisensor | iHomeLab, HSLU, Luzern CH | 0.1-10 |

Sensors used for the in-home recording

Figure 1 supplementary material.


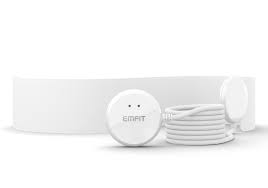

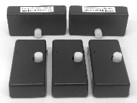

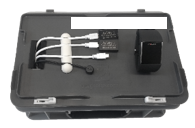

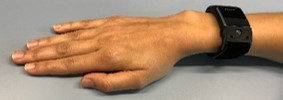


The wearable and ambient sensors used: Smart watch (top left); Set of ambient sensors (top right): they act as a Bluetooth low-energy beacon (passive); the piezoelectric bed sensor (bottom left); the base station: uploads data from the smartwatch to a server and serves as a charging station.
